# A Proactive Agent Collaborative Framework for Zero‐Shot Multimodal Medical Reasoning

**DOI:** 10.1002/aisy.202400840

**Published:** 2025-02-05

**Authors:** Zishan Gu, Fenglin Liu, Jiayuan Chen, Changchang Yin, Ping Zhang

**Affiliations:** ^1^ Department of Computer Science and Engineering The Ohio State University Columbus OH USA; ^2^ Department of Biomedical Informatics The Ohio State University Columbus OH USA; ^3^ Department of Engineering Science, Institute of Biomedical Engineering University of Oxford Oxford UK; ^4^ Translational Data Analytics Institute The Ohio State University Columbus OH USA

**Keywords:** AI agent, large language model, multimodal medical reasoning

## Abstract

The adoption of large language models (LLMs) in healthcare has garnered significant research interest, yet their performance remains limited due to a lack of domain‐specific knowledge, medical reasoning skills, and their unimodal nature, which restricts them to text‐only inputs. To address these limitations, we propose MultiMedRes, a multimodal medical collaborative reasoning framework that simulates human physicians’ communication by incorporating a learner agent to proactively acquire information from domain‐specific expert models. MultiMedRes addresses medical multimodal reasoning problems through three steps i) Inquire: The learner agent decomposes complex medical reasoning problems into multiple domain‐specific sub‐problems; ii) Interact: The agent engages in iterative “ask‐answer” interactions with expert models to obtain domain‐specific knowledge; and iii) Integrate: The agent integrates all the acquired domain‐specific knowledge to address the medical reasoning problems (e.g., identifying the difference of disease levels and abnormality sizes between medical images). We validate the effectiveness of our method on the task of difference visual question answering for X‐ray images. The experiments show that our zero‐shot prediction achieves state‐of‐the‐art performance, surpassing fully supervised methods, which demonstrates that MultiMedRes could offer trustworthy and interpretable assistance to physicians in monitoring the treatment progression of patients, paving the way for effective human–AI interaction and collaboration.

## Graphical abstract

This work proposes a multimodal medical collaborative reasoning framework, which imitates clinician's working patterns of comparative analysis. The framework includes a cohort of domain‐expert models, and an large language model learner agent to generate inquiries and interact with these experts to gather the essential information, and integrate the conversation to answer the initial multimodal reasoning question.

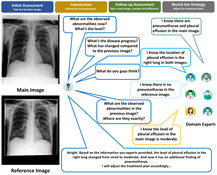

## 1 Introduction

In the domain of medical informatics, the integration of deep learning techniques with extensive hospital database resources has been rapidly evolving these years, particularly in the multimodal analysis of chest X‐ray images.^[^ ^1^, ^2^, ^3^ ^]^ This effort has attracted the attention of researchers working on various medical multimodal reasoning tasks, including the automatic generation of radiological reports,^[^ ^4^, ^5^, ^6^, ^7^ ^]^ and answering pre‐defined medical inquiries.^[^ ^8^, ^9^ ^]^ In particular, the task of difference visual question answering (DVQA) in radiology^[^ ^10^ ^]^ addresses the challenge of analyzing sequential images of the same patient taken at different times. This requires the model to answer comparative medical questions that involve assessing changes between images, for example, “What has changed compared to the previous image?” Hence, to accurately address these questions, models must not only understand each image effectively and identify abnormalities to assist in diagnosis but also precisely describe the differences between the input images, specifically, the progression of disease. Thus, DVQA in radiology is emphasized as a critical task that mirrors the real‐life diagnostic processes closely: clinicians routinely compare sequential X‐ray images of the same patient to monitor disease progression and treatment efficacy.

**Figure 1:**
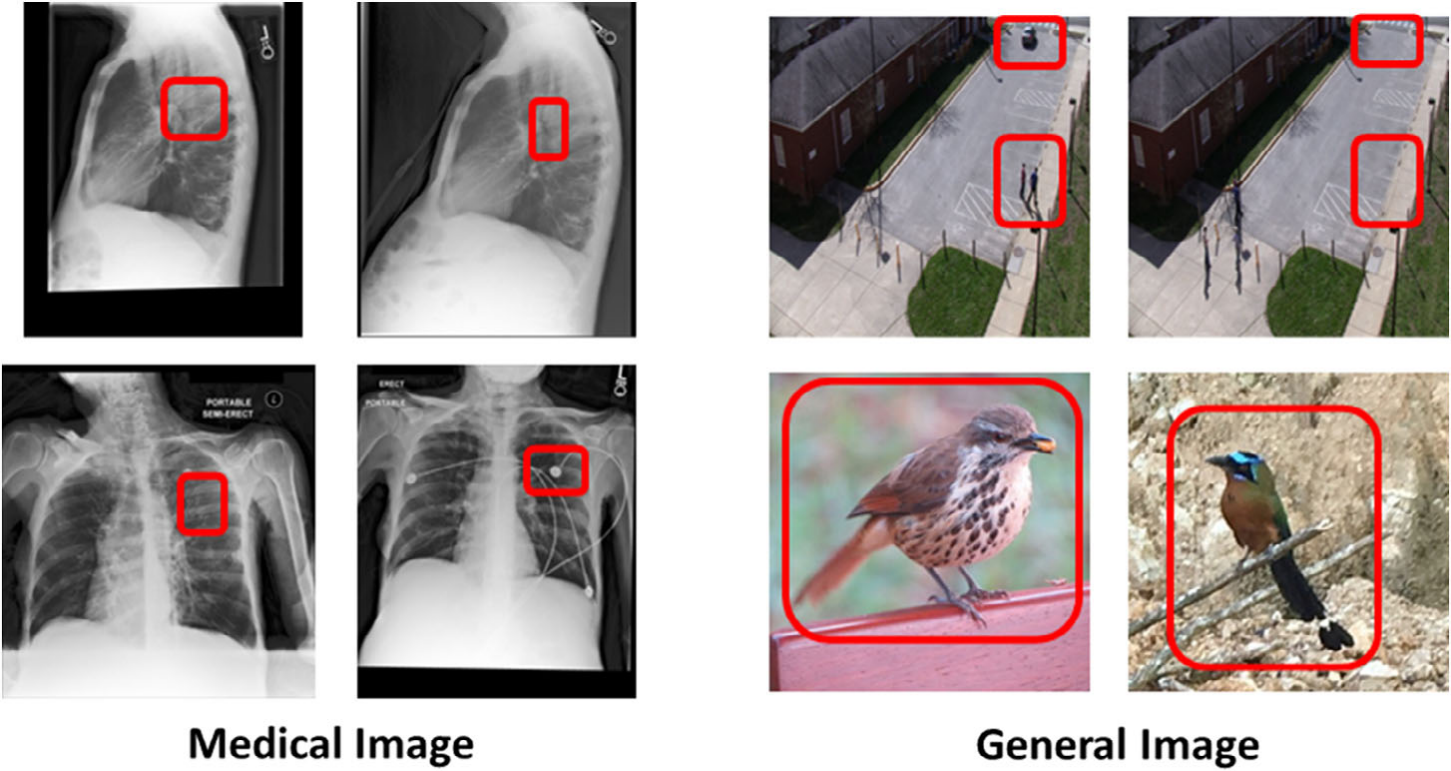
Medical image comparison versus general image comparison. In general image comparison tasks, significant differences often exist between the main subjects, or the viewpoint remains static, allowing pixel‐level comparisons before focusing on captioning. However, in medical image comparison, the main subjects are typically the same, with nuanced differences found in subtle details that require a deep understanding of medical knowledge for accurate interpretation.

Answering differences in medical images inherently poses more complexity, especially compared to general images. As illustrated in **Figure** **1**, typical DVQA tasks for general images exhibit two characteristics: different images i) may display pronounced differences in their main objects, such as in the case of two distinct birds,^[^ ^11^ ^]^ or ii) may share the same, usually fixed, viewpoints as those from a video camera, featuring significant changes in salient content.^[^ ^12^ ^]^ Consequently, clear differences in visual features between images are present, enabling the model to effectively capture their differences. However, in the context of medical imaging, on the one hand, the main objects (e.g., the abnormalities) remain identical, with sought‐after differences residing in subtle details (e.g., the severity and the size of the abnormalities). Moreover, it is the identical regions that predominantly feature across images, rather than the differing ones. As shown in Figure 1c highlighted by red bounding boxes, constitute only a minor portion of the overall images. This leads to the visual differences between medical images being relatively understated, thereby hindering the model's ability to discern differences through direct feature comparison. Instead, a model must employ a higher, more abstract level of understanding and comparison to accurately identify changes.^[^ ^10^ ^]^ On the other hand, X‐ray images of the same patient, even when taken in the same body position, can vary in viewpoints and scale, further making discrimination more difficult. Therefore, to answer differences between X‐ray images, a model necessitates a comprehensive understanding of varied domain‐specific knowledge to accurately identify a wide range of clinical findings, which may present more significant differences than those discernible through visual features alone. Furthermore, the model must also be capable of recognizing the subtle yet critical differences within the main objects (e.g., disease progress), which are pivotal for effective disease monitoring.

**Figure 2:**
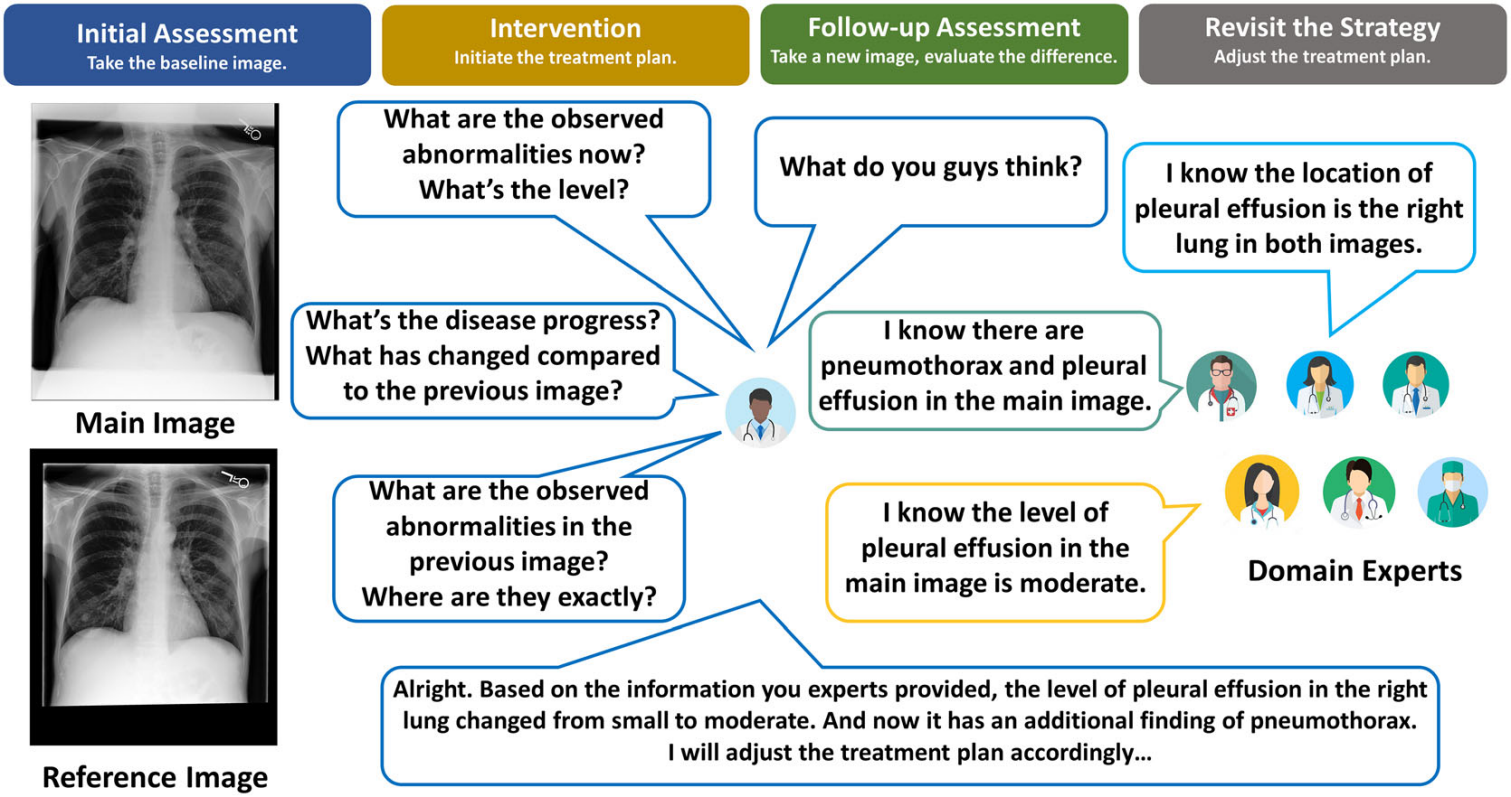
Illustration of the expert consultation in diagnosing a patient based on medical image comparison. The workflow begins with an initial assessment to capture a baseline image, followed by an intervention phase where domain experts analyze abnormalities and their progression between a reference and a follow‐up image. The experts provide detailed insights on the condition, such as pleural effusion and pneumothorax, contributing to a comprehensive diagnosis. The feedback loop continues with follow‐up assessments and strategy adjustments to optimize the patient's treatment plan.

To effectively capture domain‐specific knowledge and the subtle yet critical differences between chest X‐ray images, we propose a multimodal medical collaborative reasoning framework MultiMedRes, which imitates the clinicians’ working patterns. As shown in **Figure** **2**, the common practice of a patient treatment process^[^ ^13^ ^]^ typically commences with the acquisition of a baseline image to serve as diagnostic evidence and the foundation for initiating a treatment plan. Then a follow‐up image is obtained to monitor and assess the intervention's efficacy throughout the treatment period. This process often involves consultations with multiple experts across various domains, which may lead to modifications of the initial treatment plan. To model the above working patterns, MultiMedRes introduces three steps led by a learner agent: i) Inquire: The learner agent aims to understand the broad challenging difference questions and ask multiple types of domain‐specific sub‐questions that target specific aspects of the images, for example, detecting abnormalities, identifying the level of severity and the location of the disease; ii) Interact: The learner agent feeds the sub‐questions into expert models (i.e., specialists) to obtain their answers. These expert models are pre‐trained on the domain‐specific task and data to store rich specific knowledge for each type of domain‐specific question. Our method will then raise new questions based on the given answers. Through repeating the “ask‐answer” interaction process, our agent can progressively obtain sufficient knowledge for different domain‐specific questions from the expert models; and iii) Integrate: The learner agent finally integrates all knowledge from the domain‐specific specialists to address the input difference question accurately. These three steps can also provide readily interpretable information for human radiologists.

We conduct the experiments on the benchmark MIMIC‐Diff‐VQA^[^ ^10^ ^]^ dataset. Experiments demonstrate that our approach can achieve state‐of‐the‐art performances. Moreover, MultiMedRes can be incorporated into diverse LLMs and multimodal LLMs to significantly boost their performance. The contributions of our study are outlined as follows: 1) We introduce a collaborative reasoning framework, MultiMedRes, which enables an LLM learner agent to acquire essential domain‐specific knowledge from specialized expert models to perform zero‐shot medical multimodal reasoning. 2) The zero‐shot prediction provided by our framework achieves state‐of‐the‐art performance, and even outperforms the fully supervised methods, which demonstrates that MultiMedRes has the potential to provide trustworthy and interpretable assistance to physicians in monitoring the treatment progression of patients, paving the way for effective human–AI interaction and collaboration. 3) Experiments demonstrate that our framework is compatible with and can be easily generalized to various LLMs, including both text‐based LLMs (e.g., GPT‐3.5 and GPT‐4) and multimodal LLMs (e.g., LLaVa).

## 2 Overall Framework

**Figure 3:**
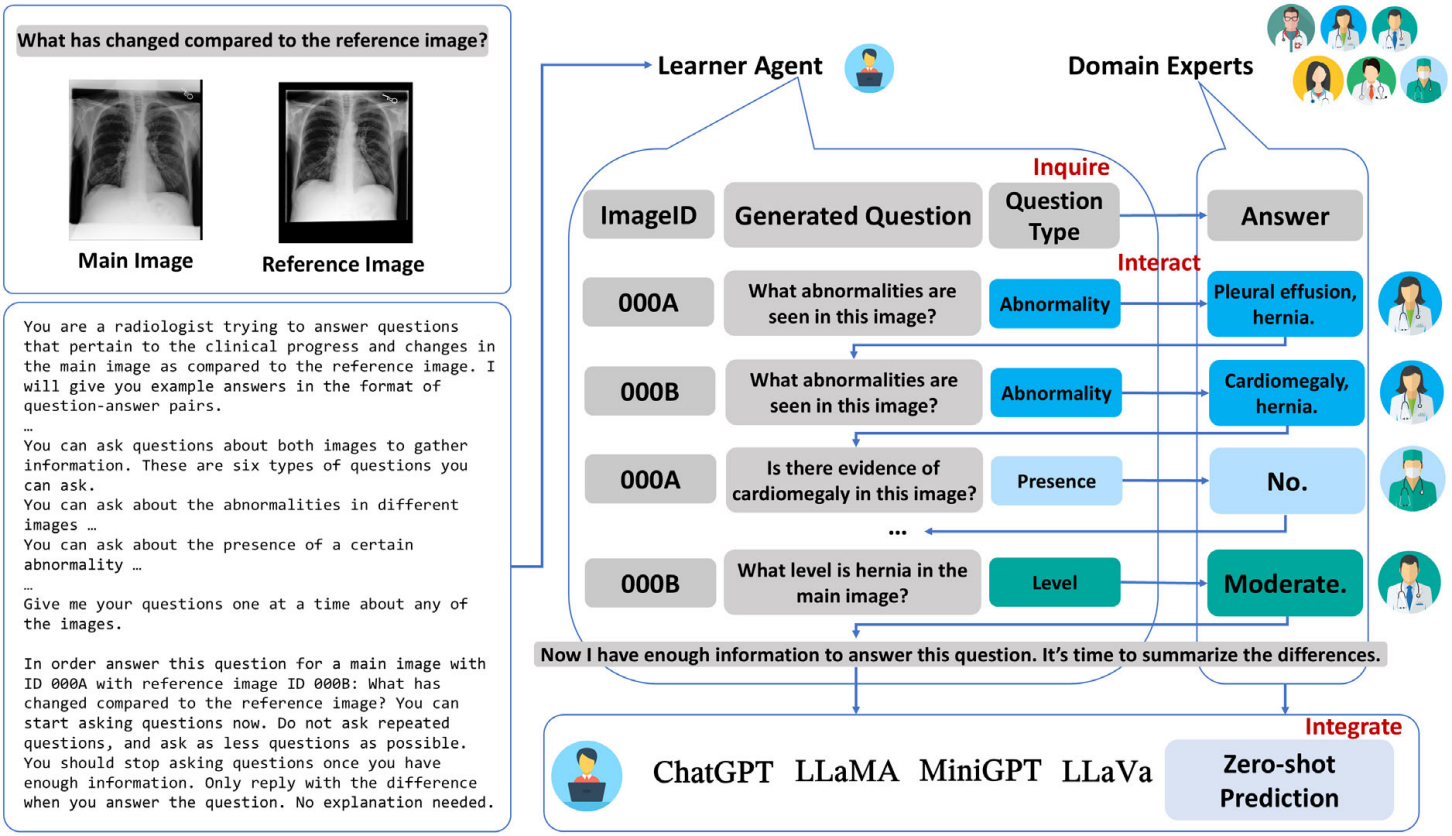
The proposed MultiMedRes framework. Upon receiving questions comparing two images, the learner agent employs an iterative approach, generating questions related to either the main image or the reference image, before consulting the appropriate domain expert (i.e., specialists). Upon collecting sufficient information, the learner agent is prompted to cease question generation and integrate the information to provide a zero‐shot prediction.

**Figure** **3** displays the overview of the proposed MultiMedRes, a novel collaborative framework for medical reasoning tasks related to medical imaging. In this study, we mainly focus on the challenging yet essential medical reasoning task: difference question answering (i.e., DVQA). This type of questions inquire about the comparison between current and previous images of the same patients and studies the treatment or disease progress, which is more consistent with real‐life radiologists’ practice. As illustrated in Figure 3, our framework would take two images and a question regarding both images as input and output a generated text captioning the difference. More specifically, we first train a cohort of domain‐expert models, each tailored to address a distinct category of questions (e.g., abnormality detection, localization), to serve as domain‐specific experts. Subsequently, an LLM learner agent is designed to generate inquiries and interact with these experts to gather the essential information for the medical reasoning questions, for example, difference questions. Finally, once the agent obtains enough information, it will stop asking questions and integrate the conversation for answering the initial difference inquiry.

## 3 Results

### 3.1 Dataset

In this study, we conduct comprehensive experiments on the newly released DVQA dataset of chest X‐ray images, MIMIC‐Diff‐VQA,^[^ ^10^ ^]^ which was derived from the open benchmark dataset MIMIC‐CXR.^[^ ^2^ ^]^ It includes a total of seven types of questions, with six types being questions regarding single images and the last type with 164 324 questions that require the comparison of two images, referred to as *difference* questions. We adhered to the original data splits with a train/validation/test ratio of 8/2/2. To maintain the integrity of the evaluation process, there is no overlap of images across the different sets (train, validation, and test). For example, if an image is used for one type of question in the training set, it will appear exclusively in the training set for other question types as well, ensuring no test images are leaked into the training set for domain experts. The dataset's statistics and two examples for each question type are provided in Table S1, Supporting Information, which also serve as context‐learning material for LLM learner agents.

### 3.2 Experimental Results

#### 3.2.1 Main Results

**Figure 4:**
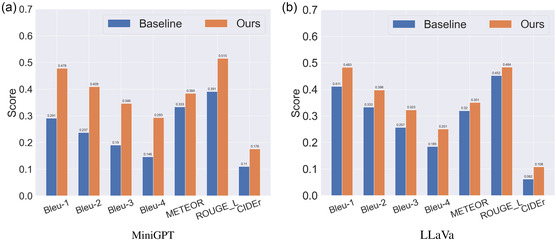
Performance comparison for vision LLMs with or without our MultiMedRes. a) Results for MiniGPT. b) Results for LLaVA. As we can see, our method significantly boosts the performance of vision LLMs across all metrics.

We report the models’ performance with difference questions in **Table** **1**, and the performance of expert models on questions regarding single images in Table S3, Supporting Information. It can be observed that, training the EKAID model solely with difference questions results in a moderate improvement in performance compared to the baseline established by the original work. As for the general‐purpose large‐scale multimodal models, specifically Unified‐IO,^[^ ^14^ ^]^ MiniGPT‐v2,^[^ ^15^ ^]^ and LLaVa,^[^ ^16^ ^]^ they all struggle to attain competitive performance on both DQA task and VQA task, likely attributable to a deficiency in domain knowledge. Moreover, while medical domain‐specific models like LLaVa‐Med^[^ ^17^ ^]^ and LLM‐CXR^[^ ^18^ ^]^ demonstrate better performance in VQA for single‐image tasks, their performance in the few‐shot setting for DVQA is even lower than that of LLaVa. This is likely due to their limited domain‐specific fine‐tuning, which constrains their generalizability to novel tasks where the input consists of two images. Conversely, leveraging the significantly enhanced capabilities of domain‐expert models, facilitated by our divide‐and‐conquer strategy, the zero‐shot predictions generated by GPT‐3.5, LLaMa2‐70B, and GPT‐4V and GPT‐4 through our proposed MultiMedRes all demonstrate competitive performance. Notably, GPT‐4 significantly surpasses the previous state‐of‐the‐art models, including the fine‐tuned domain‐specific LLM and the fully supervised generative model, without requiring specific training on DVQA data. GPT‐4 V also achieves comparable results as the learner agent, with only one out of the six evaluation metrics showing a slight improvement over the pure text‐based version. We interpret this outcome as a result of GPT‐4V's limited visual knowledge specific to chest X‐ray images, which restricts its ability to provide more accurate interpretations from visual inputs. This result indicates that, it is the strong reasoning ability of the text‐based agent that plays a pivotal role in the success of MultiMedRes. Furthermore, we incorporate the dialogue between the learner agent and the specialists into the prompts for vision LLMs, such as MiniGPT‐v2 and LLaVa. The differences between prompts with and without our generated dialog are illustrated in Figure S4, Supporting Information. As shown in **Figure** **4**, this integration significantly enhances their performance. This improvement, combined with the outstanding results from the zero‐shot prediction capability of MultiMedRes, suggests that the dialog between the learner agent and the specialists indeed encapsulates essential domain knowledge absent in the LLMs, shedding light on the adaptive usage of LLMs in specific domains.

**Table 1:** Comparative performance of various models on the difference question answering task. EKAID and EKAID_diff represent the state‐of‐the‐art task‐specific models, which adopt the labeled data to perform fully supervised training. The last three rows represent the zero‐shot performance of MultiMedRes with the learner agent being GPT‐3.5, LLaMa2, GPT‐4V, and GPT‐4 respectively. Bold numbers indicate the best performance for each metric.

Models	Bleu‐1	Bleu‐2	Bleu‐3	Bleu‐4	METEOR	ROUGE_L	CIDEr
EKAID^[^ ^10^ ^]^	0.569	0.498	0.438	0.382	0.304	0.547	0.823
EKAID_diff^[^ ^10^ ^]^	0.606	0.529	0.468	0.410	0.350	0.572	0.827
UIO^[^ ^14^ ^]^	0.360	0.309	0.267	0.223	0.220	0.426	0.388
MiniGPT‐v2^[^ ^15^ ^]^	0.291	0.237	0.190	0.146	0.333	0.391	0.110
LLaVa^[^ ^16^ ^]^	0.411	0.333	0.257	0.185	0.320	0.452	0.162
Med‐Flamingo^[^ ^22^ ^]^	0.573	0.505	0.433	0.359	0.304	0.544	0.438
LLaVa‐Med^[^ ^17^ ^]^	0.366	0.295	0.225	0.125	0.224	0.420	0.190
XrayGPT^[^ ^28^ ^]^	0.102	0.037	0.015	0.006	0.089	0.124	0.056
LLM‐CXR^[^ ^18^ ^]^	0.161	0.093	0.062	0.038	0.072	0.153	0.128
MultiMedRes (GPT‐3.5)	0.497	0.423	0.361	0.303	0.317	0.526	0.451
MultiMedRes (LLaMa2)	0.537	0.465	0.391	0.345	0.373	0.554	0.483
MultiMedRes (GPT‐4 V)	0.599	0.526	0.466	0.412	**0.375**	0.573	0.833
MultiMedRes (GPT‐4)	**0.610**	**0.535**	**0.473**	**0.418**	0.357	**0.586**	**0.843**

#### 3.2.2 Augmented Training Data

**Figure 5:**
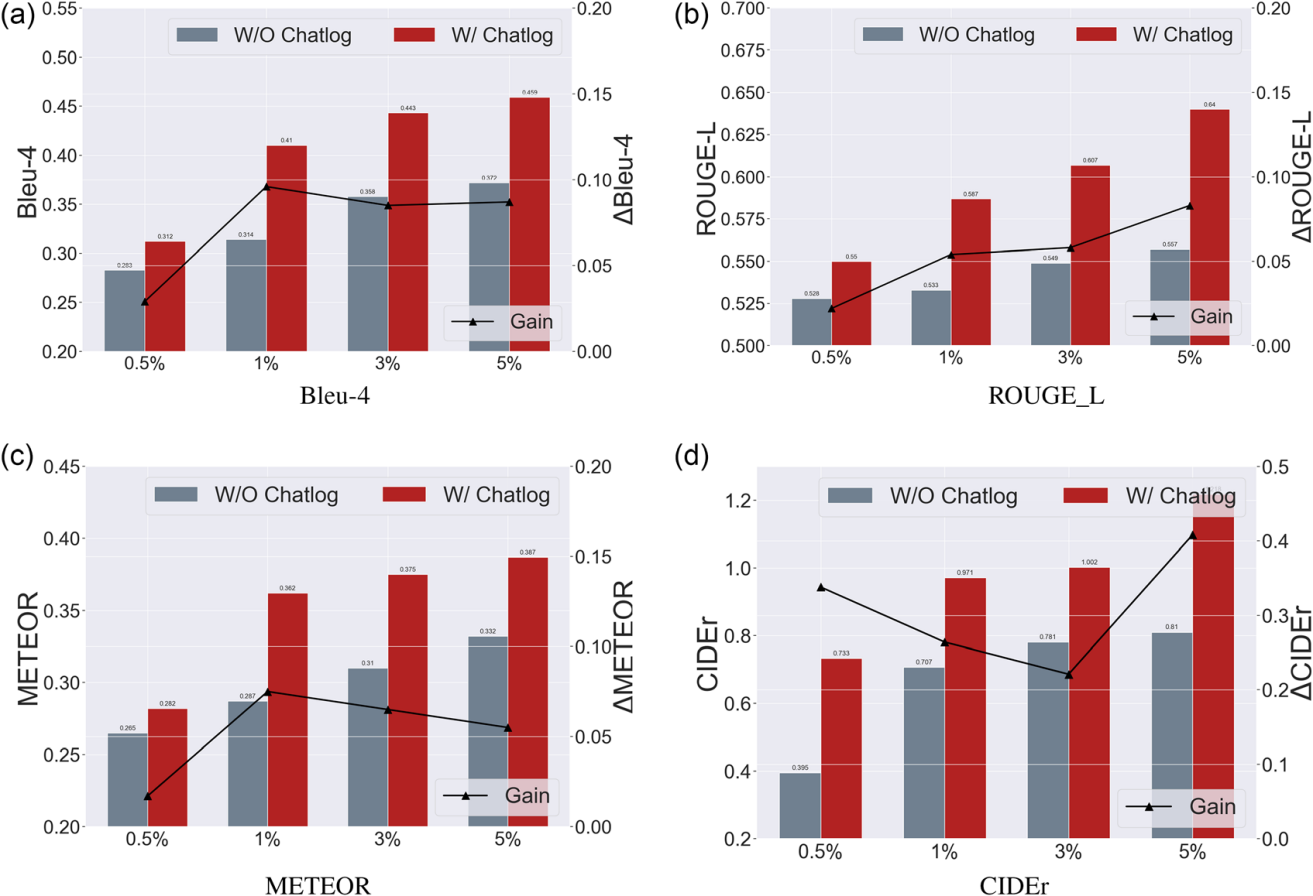
Bleu‐4, METEOR, ROUGE_L and CIDEr score of the few‐shot prediction generated by EKAID with respect to various ratios of labeled data (i.e., question–answer pairs) for training. The comparison is between the model trained with or without the augmented training data. a) Bleu‐4 scores, b) ROUGE_L scores, c) METEOR scores, and d) CIDEr scores. The differences at different ratios are depicted using a polyline and the scales are indicated on the right y‐axis.

To demonstrate the generalizability of our proposed framework, we further incorporate our generated conversation between the learner agent and the specialists as part of the training data for the supervised model EKAID. Specifically, instead of only considering the difference question as the text input, we also input the conversation chatlog for the training and test samples. We present the performance of EKAID with varying proportions of the training set in **Figure** **5**. It is observed that our generated chatlog enables the model to consistently outperform one trained without it, and it further improves its performance with an increase in training data. Notably, with only 1% of training data, the EKAID model with augmented training data achieves comparable performance with the one trained with all of the training data without chatlog. With 5% of the training data, it also outperforms the zero‐shot prediction generated by the MultiMedRes. This is because the enhancement of zero‐shot learning benefits from, and at the same time, is limited by the answers provided by the domain‐expert models. In other words, its performance is largely determined by the answer accuracy of questions regarding single images. On the contrary, while LLMs tend to accept whatever the specialists return and include everything in their answers, the supervised few‐shot prediction model with augmented training data has the opportunity to identify and correct the incorrect, retaining only the essential information in the final answer. We will further discuss this difference in the following paragraphs.

### 3.3 Conversation Study

**Figure 6:**
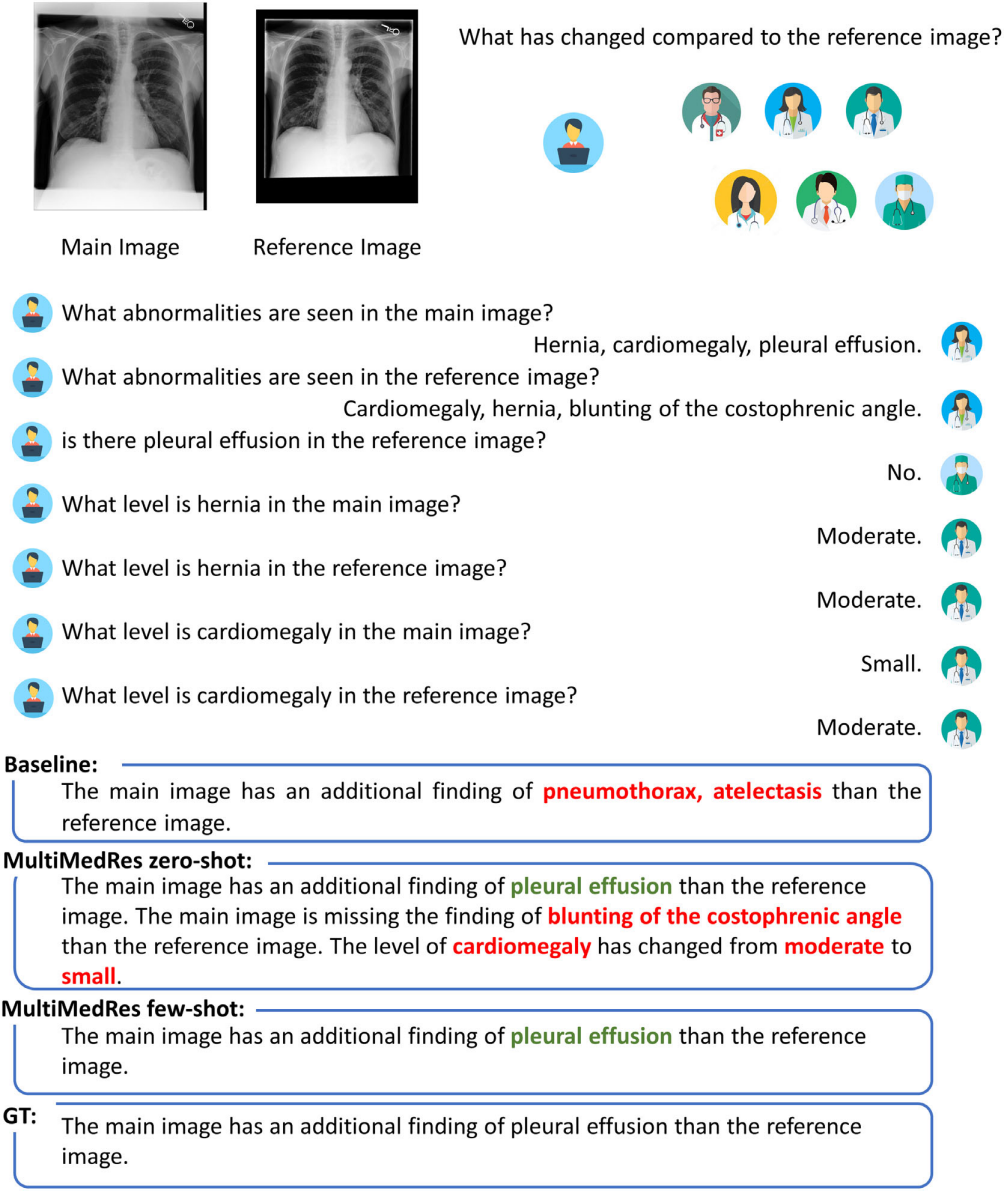
Case study for the commonly seen question: “What has changed compared to the reference image?” We highlight the same information mentioned in the ground truth answer with the color green, and the redundant or incorrect information with the color red.

**Figure 7:**
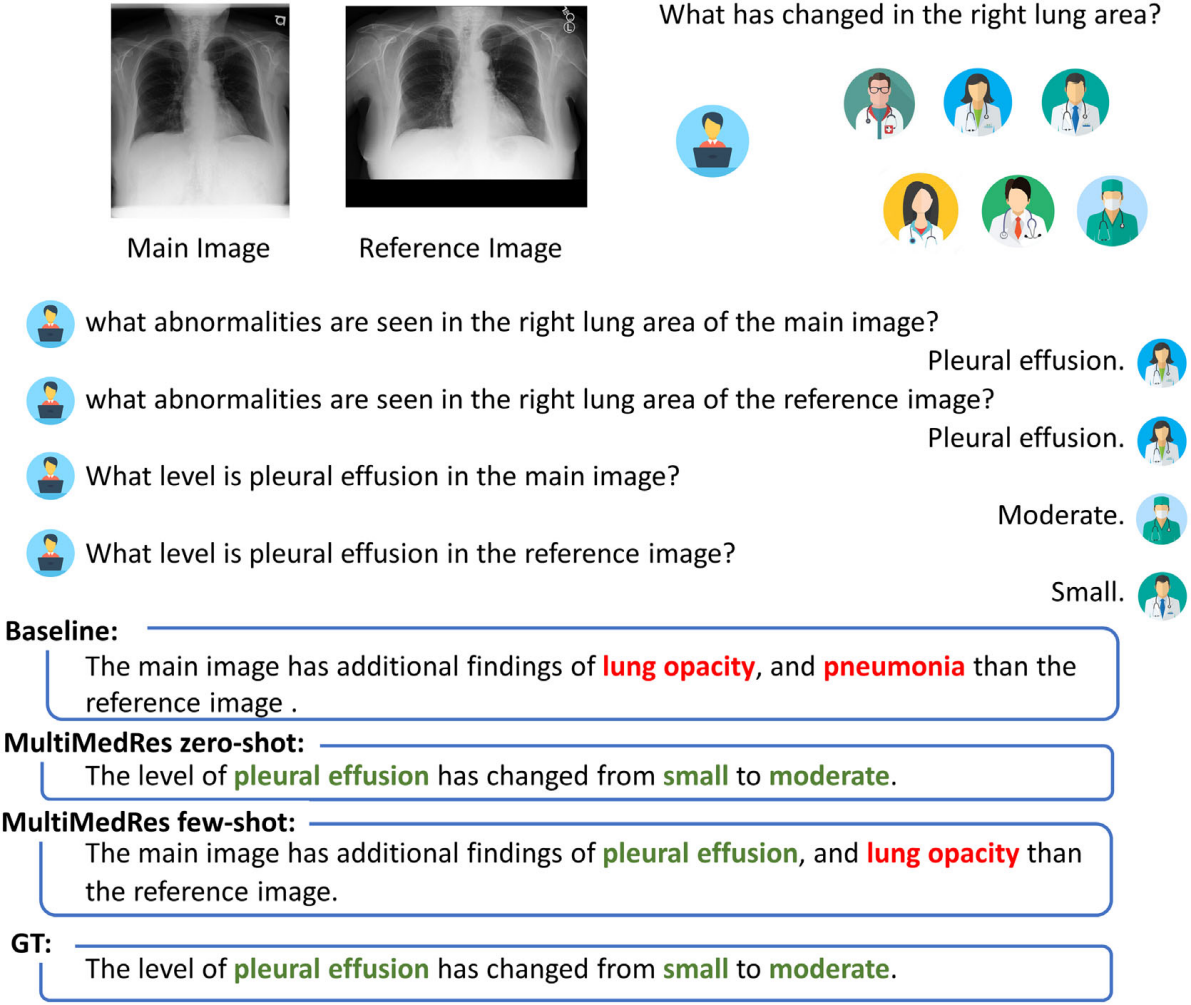
Case study for the relatively rare question: “What has changed in the right lung?” We highlight the same information mentioned in the ground truth answer with the color green, and the redundant or incorrect information with the color red.

We present two case studies in **Figure** **6** and **7** to more effectively illustrate our proposed framework. In addressing the common general question, “What has changed compared to the reference image?”, as depicted in Figure 6, the learner agent initially asks about image abnormalities from a global perspective, responses to which are provided by the abnormality detection specialist. Upon identifying the abnormalities in both images, the learner agent then consults the abnormality level specialist to examine the severity of recurring abnormalities, such as *hernia* and *cardiomegaly* in this instance, discovering a change in the level of *cardiomegaly*. Finally, having gathered sufficient information, the agent concludes its inquiries and formulates a comprehensive response incorporating all discussed information. However, this approach may not always yield the most accurate results. For instance, the specialists’ responses might occasionally be inaccurate or highlight irrelevant details, as shown in Figure 6. The change in *cardiomegaly* severity from small to moderate may be less critical than the new finding of *pleural effusion*, and the noted *blunting of the costophrenic angle* might be a false positive. Thus, including all these details, as the learner agent does, could lead to worse performance. Conversely, a supervised few‐shot prediction model can potentially rectify these inaccuracies with direct access to the images. This improvement is contingent on the inclusion of chatlog as input, which enables the model to efficiently identify the correct information through the conversation, as opposed to extracting it by itself. With the chatlog, the search scope for the supervised model is significantly narrowed, yielding enhanced performance with a reduced reliance on training data.

It is worth noticing that the LLM learner agent can also rectify errors made by the specialists. In our observations, the learner agent occasionally poses what appears to be a redundant question, such as verifying the presence of a disease previously indicated by the abnormality specialist, or mentioning an abnormality not identified earlier in the discussion. Interestingly, these additional queries may lead the specialists to reconsider their previous statement, potentially leading to a more accurate final answer. We hypothesize that such questions derive from the extensive knowledge base of the LLMs. For instance, if the LLM rarely encounters instances of *vascular congestion* co‐occurring with *cardiomegaly* in prior radiology reports, it might seek verification from the specialists, thereby potentially rectifying any inaccuracies. Indeed, addressing a simple yes‐or‐no question about a specific abnormality is significantly more straightforward than identifying all abnormalities, and it is evident that our specialists respond more accurately to these direct inquiries, as evidenced in Table S3, Supporting Information. Similarly, we illustrate such a rectifying process instance in Figure S1, Supporting Information. Additionally, for less common inquiries such as “What has changed in the right lung?” shown in Figure 7, the LLM learner agent skillfully tailors its questions to acquire more targeted information. In these scenarios, the zero‐shot answer from the learner agent is often more relevant, since the supervised few‐shot answer may default to more general response patterns due to limited exposure to such uncommon cases. Given this analysis, the LLM learner agent is undoubtedly an integral component of our proposed framework. And, more importantly, our MultiMedRes is equipped to effectively harness its reasoning abilities and extensive knowledge base to tackle specific domain tasks.

## 4 Discussion

### 4.1 Bias Evaluation

In this section, we assess the DVQA performance of our approach compared to the previous state‐of‐the‐art, utilizing more fine‐grained datasets. Specifically, we further segregate our test set into two subsets based on gender and age. To ensure a relatively even distribution of data and adhere to medical intuition, we categorize ages into three groups: Age<55(29.1%), 55≤Age<70(33.9%), and Age≥70(37.0%). The results are presented in **Table** **2**. As observed, the prior method, EKAID, exhibits substantial bias when encountering different age and gender groups, achieving significantly better performance toward elder patients and male patients. Conversely, both the zero‐shot predictions made by LLMs and the few‐shot predictions made by EKAID with augmented training data effectively reduce such bias. This demonstrates the generalization capability and effectiveness of our method across diverse datasets and patient groups.

**Table 2:** Comparison of baseline against MultiMedRes for two different kinds of split. Bold numbers indicate the best performance for each metric.

Gender	Ratio of Data	Female										Male								
		Bleu‐4		METEOR			ROUGE_L			CIDEr		Bleu‐4		METEOR			ROUGE_L		CIDEr	
EKAID	100%	0.398		0.342			0.556			0.805		0.417		0.357			0.583		0.860	
MultiMedRes	0%	0.414		**0.358**			0.579			0.830		0.416		0.355			0.595		0.858	
	5%	**0.454**		0.356			**0.596**			**1.261**		**0.457**		**0.364**			**0.608**		**1.141**	
Age	Ratio of Data	Age<55					55 ≤Age<70							70 ≤ Age						
		Bleu‐4	METEOR		ROUGE_L	CIDEr		Bleu‐4	METEOR		ROUGE_L		CIDEr		Bleu‐4	METEOR		ROUGE_L		CIDEr
EKAID	100%	0.364	0.350		0.537	0.887		0.426	0.352		0.585		0.849		0.445	0.348		0.597		0.743
MultiMedRes	0%	0.406	0.356		**0.597**	0.968		0.418	0.360		0.581		0.847		0.424	0.356		0.575		0.676
	5%	**0.432**	**0.358**		0.586	**1.472**		**0.467**	**0.362**		**0.609**		**1.096**		**0.471**	**0.362**		**0.619**		**0.915**

### 4.2 Human Evaluation

We have conducted a human evaluation experiment to demonstrate the efficacy of our proposed framework. Specifically, we invite two professional physicians to evaluate the perceptual quality of 100 randomly selected difference questions and the answers generated by EKAID (fully supervised model), Med‐Flamingo (fine‐tuned visual LLM) and our MultiMedAgent (Zero‐shot). To ensure objectivity, the evaluators were blinded to the origin of the answers and were asked to identify the response most closely aligning with the Ground Truth (GT) answer, or to declare a tie if appropriate. The results, as detailed in **Table** **3**, reveal that MultiMedRes significantly outperforms the baseline models in a clinical setting, as evidenced by its highest pick‐up percentages. This finding affirms that the integration of LLM learner agents with domain‐specific expert models not only leverages the strengths of both approaches but also substantially enhances the system's reliability and clinical applicability.

**Table 3:** Human evaluation with 100 randomly selected questions and the answers generated by EKAID (fully supervised model), Med‐Flamingo (fine‐tuned visual LLM), and our MultiMedAgent (zero‐shot). We ask the evaluators to identify the response most closely aligning with the ground truth (GT) answers, and highlighted the highest pick‐up percentages.

EKAID wins	Med‐Flamingo wins	MultiMedRes (Zero‐shot) Wins	Tie
21	12	**48**	19

### 4.3 Ablation Study

We have conducted an ablation study to evaluate the impact of the divide‐and‐conquer strategy and the integration of an abnormality detection specialist within our framework. As shown in **Table** **4**, the performance for incorporating both LLaMa2 and ChagtGPT‐4 as the learner agent drops without any of these extra modules. The results underscore that both the divide‐and‐conquer approach and the inclusion of the abnormality detection specialist significantly contribute to the enhanced performance of our framework. redAdditionally, we incorporate LLM‐CXR, the best‐performing domain‐specific model, as a domain expert in Table 4. The results reveal that, with less accurate answers to questions involving single‐image inputs, it fails to achieve performance comparable to our proposed MultiMedRes, further demonstrating the superiority of our approach.

**Table 4:** Abaltion study of the divide‐and‐conquer strategy and the integration of an abnormality detection specialist with LLaMa2 and ChagGPT‐4 being the learner agent.

Learner Agent	Model variation	Bleu‐4	METEOR	ROUGE_L	CIDEr
LLaMa2‐70b‐chat	w/o divide‐and‐conquer	0.299	0.321	0.495	0.412
	w/o abnormality detection	0.320	0.356	0.532	0.456
	w/LLM‐CXR	0.326	0.302	0.486	0.435
	–	0.345	0.373	0.554	0.483
ChatGPT‐4‐Turbo	w/o divide‐and‐conquer	0.331	0.313	0.487	0.637
	w/o abnormality detection	0.408	0.351	0.564	0.806
	w/LLM‐CXR	0.373	0.297	0.516	0.703
	–	0.418	0.357	0.586	0.843

## 5 Experimental Section

In this section, we illustrate our proposed MultiMedRes, a novel collaborative framework for medical reasoning tasks. Initially, we train a cohort of domain‐expert models, each tailored to address a distinct category of questions (e.g., abnormality detection and localization), to serve as domain‐specific experts. Subsequently, an LLM learner agent is designed to generate inquiries and interact with these experts to gather the essential information for the medical reasoning questions. Finally, once the agent obtains enough information, it will stop asking questions and integrate the conversation for answering. Figure 3 displays the overview of the proposed MultiMedRes.

### 5.1 Problem Statement

For common medical questions involving a single image (VQA), we adhere to the standard practice of VQA tasks by implementing classification models. Our trained domain‐expert models, denoted as ${ℱ}_{\text{sp}}$, are designed to receive an image i∈I and a question q∈Q as inputs, and to subsequently predict the label a∈A, which corresponds to answers observed in the dataset. Further, in this study, we mainly focus on the challenging yet essential medical reasoning task: difference question answering (i.e., DVQA). This type of questions inquires about the comparison between current and previous images of the same patients and studies the treatment or disease progress, which is more consistent with real‐life radiologists’ practice. As illustrated in Figure 3, our framework would take two images $i_{1},i_{2}\in I$ and a difference question $q_{d}\in Q$ as input and output the difference captioning regarding the question. More specifically, the agent ${ℱ}_{\text{learner}}$ will generate questions concerning single images iteratively and interact with the corresponding specialist models to derive an answer in each round of the conversation, terminating the process upon acquiring sufficient information to formulate a response to the initial difference question.

In the subsequent section, we aim to design our prompting system to assist the LLM agent ${ℱ}_{\text{learner}}$ in comprehending the task, formulating the appropriate questions, and concluding the conversation appropriately.

### 5.2 Learner Agent

In MultiMedRes, we introduce an automatic question generation mechanism that leverages LLM's in‐context learning and reasoning abilities. We utilize the LLM as a learner agent to generate questions as well as the corresponding question types about chest X‐ray images, and direct these questions to well‐trained domain‐expert models for answer retrieval.

Mimicking the reasoning process of a human being when answering a difference question, a learner agent must first gather information of the main image and the reference image individually, and then proceed to summarize the difference. Specifically, we incorporate ChatGPT^[^ ^19^ ^]^ or LLaMa^[^ ^20^ ^]^ as the learner agent responsible for generating questions. To optimize the in‐context learning and reasoning abilities of the LLMs, we design the prompting system with three parts: a systematic instruction $\rho_{\text{task}}$ to describe the difference question–answering task, a context‐learning instruction $\rho_{Q}$ to guide question generation, and an appended instruction $\rho_{i}$ to signal the end of the questioning process and initiate summarization. Note that during the conversations, the generated question–answer pairs in earlier rounds (denoted as $\rho_{\text{log}}$) are also visible to the agent. Consequently, every question is generated using the combined prompt of $\rho_{\text{task}}+\rho_{Q}+\rho_{i}+\rho_{log}$.

#### 5.2.1 Task Instruction $\rho_{\text{task}}$

The task instruction $\rho_{\text{task}}$ outlines the task that the learner is required to perform and provides the material for in‐context learning of the difference question answering. It guides the LLM to utilize the related knowledge base and generate questions to gather information on images to compare the differences. $\rho_{\text{task}}$ is designed as follows:

*You are a radiologist trying to answer questions that pertain to the clinical progress and changes in the main image as compared to the reference image. I will give you example answers in the format of question–answer pairs. [context] Please answer this question for a main image A with reference image B: [difference questions]*

The initial sentence directs the learner agent to access and apply radiology‐related knowledge bases, while the context will demonstrate the desired answer formats (as shown in Figure S2 and S3, Supporting Information).

#### 5.2.2 Question Generation Instruction $\rho_{Q}$

To assist the learner agent in question generation, we supply both the categories and contents of the questions, which facilitate the identification of the most appropriate domain‐expert model. $\rho_{Q}$ is structured as follows:

*You can ask questions about both images to gather information, but do not ask redundant questions. You can ask about the abnormalities in different images, like […]. You can ask about the presence of a certain abnormality in an image, like […]. You can ask about the level of a certain abnormality in an image with […]. Give me your questions one at a time about any of the images. Only return the generated question, the question type, and the corresponding image ID.*

[…] above represents the corresponding question format of the given types, which will guide the LLM to generate the desired questions.

#### 5.2.3 Appended Instruction $\rho_{i}$

From an efficiency perspective, the agent should stop asking questions once it gathers enough information, and answer with simplicity. Thus, $\rho_{i}$ as the last part of the prompt is structured as follows:

*You should answer the question like the previous examples once you have enough information. Do not make any assumptions by yourself. Only reply with the difference when you answer the question. No explanation is needed.*

Please refer to Figure S3, Supporting Information for a full version of the finalized prompt.

### 5.3 Domain Expert Models

Despite the significant efforts toward developing medical vision LLMs recently, the performance of these models on specialized medical VQA datasets has failed to surpass that of the traditional state‐of‐the‐art classification models. Consequently, in this work, we continue to rely on classification models as the domain experts. However, take MIMIC‐DIFF‐VQA^[^ ^10^ ^]^ dataset shown in Table S1, Supporting Information as an example, there are totally over 9000 answer candidates for all types of questions, not including those for difference questions. This vast number of labels could potentially overwhelm a classification model, particularly when the candidates are only marginally distinct from one another. Moreover, the amount of potential answers for abnormality‐related questions is enormous as over 8000. This is particularly evident in responses to the question, “What abnormalities are seen in this image?”, where answers comprise various combinations and permutations of 33 abnormalities, further complicating the differentiation process among these answers. For example, the answer “atelectasis, penumothorax” and the answer “penumothorax, atelectasis” are the same thing, and they are only slightly different than “penumothorax, atelectasis, and pleural effusion”.

To mitigate this challenge, our framework employs a divide‐and‐conquer strategy. Specifically, we develop a distinct expert model for each question type. As in MIMIC‐DIFF‐VQA, we will train an expert model for the “Abnormality”, “Presence”, “View”, “Location”, “Type” and “Level” type of questions separately as shown in the 2nd and 7th rows in Table S1, Supporting Information. This approach significantly narrows the scope of potential answers, enabling these specialists to concentrate exclusively on their respective domains. Moreover, regarding the “abnormality” question, which essentially involves detecting abnormalities throughout the entire image, we shift our strategy. Rather than trying to match answers from an extensive training set containing a huge amount of responses, we focus on creating an abnormality detection model tailored specifically to this task, akin to multi‐label classification. This model processes the image input and generates a list of identified abnormalities, as exemplified in the chest X‐ray images. The response to this question is then formed by simply concatenating the predicted labels. Consequently, in MIMIC‐DIFF‐VQA, the number of possible answers for subsequent “abnormality” questions is reduced to 25, a volume well‐suited for a classification model.

### 5.4 Baseline Models

In this work, we consider a state‐of‐the‐are medical VQA model, MMQ,^[^ ^21^ ^]^ a state‐of‐the‐art medical DVQA model, EKAID,^[^ ^10^ ^]^ a medical domain large vision‐language model, med‐flamingo,^[^ ^22^ ^]^ and three multi‐modal LLMs UNIFIED‐IO,^[^ ^14^ ^]^ MiniGPT‐v2^[^ ^15^ ^]^ and LLaVA^[^ ^16^ ^]^ as baselines.

#### 5.4.1 MMQ

MMQ^[^ ^21^ ^]^ is a recently proposed VQA model designed for medical images, which adopts Model Agnostic *Meta*‐Learning (MAML) through pretraining multiple *meta*‐models on natural images and fine‐tuning on the medical images.

#### 5.4.2 EKAID

EKAID^[^ ^10^ ^]^ aligns the high dimensional feature of different X‐ray images through an expert knowledge graph. The model is designed to adaptively choose either to focus on the subtractive difference feature between two images or the main image feature only by utilizing the attention mechanism.

#### 5.4.3 UIO

UNIFIED‐IO^[^ ^14^ ^]^ (UIO) is a unified vision model designed to handle a broad range of vision‐language tasks, including VQA, by standardizing diverse inputs and outputs into a sequence of tokens, which enables the model to be trained on over 90 different datasets using a unified transformer‐based architecture.

#### 5.4.4 MiniGPT‐v2

MiniGPT‐v2^[^ ^15^ ^]^ is a unified LLM designed to efficiently handle a variety of vision‐language tasks based on Llama2. By using unique identifiers for different vision‐language tasks during training, the model achieves strong performance on multiple benchmarks.

#### 5.4.5 LLaVa

LLaVA,^[^ ^16^ ^]^ representing a popular practice for training multi‐modal LLMs, combines a vision encoder with a language model, which utilizes GPT‐4 to generate language‐image instruction data for improving the zero‐shot capabilities. We incorporate LLava‐v1.5 as a baseline in this work.

#### 5.4.6 Med‐Flamingo

Med‐Flamingo^[^ ^22^ ^]^ is a multimodal few‐shot text generator adapted from OpenFlamingo‐9B. It is pre‐trained on paired and interleaved image‐text data from medical publications and textbooks.

### 5.5 Agent Behavior

As illustrated in Figure 3, for questions involving the comparison of two images, or difference questions, our proposed MultiMedRes follows an iterative pattern. During the initial iteration, the learner agent formulates a query for the expert models based on the question only. For the following iterations, the agent not only considers the difference questions but also integrates information already acquired from earlier conversations. This strategy enables the LLM agent to generate the next question accordingly and determine the appropriate time to cease inquiries, thereby avoiding the generation of redundant or irrelevant questions.

### 5.6 Experiment Setting

We employ both GPT^[^ ^19^ ^]^ accessed through the OPENAI API (https://openai.com/) and LLaMa 2^[^ ^20^ ^]^ via the Replicate API (https://replicate.com/) as the learner agents. For the domain‐expert models, we integrate MMQ^[^ ^21^ ^]^ for the VQA tasks and DenseNet^[^ ^23^ ^]^ for abnormality detection. More specifically, we incorporate gpt‐3.5‐turbo‐0125, gpt‐4‐1106‐preview, and LLaMa2‐70B‐chat as the learner agents. To enhance the reproducibility and stability of their performance, we set the temperature parameter at 0.2 for both LLMs. In terms of specialist agents, we integrated the MMQ^[^ ^21^ ^]^ as the domain‐expert VQA model and a 121‐layer DenseNet^[^ ^23^ ^]^ as the multi‐label prediction model for abnormality detection. Regarding the training hyperparameters, we conducted a grid search to optimize the configurations, finding that a feature dimension of 64 and a learning rate of 0.01 for the MMQ model slightly outperformed the other setups. Details of these preliminary experiments are presented in Table S2, Supporting Information. For other settings, such as the number of pre‐trained modules and the training loss, we adhered to the configurations outlined in the original studies and retained their default values. We train expert models and perform evaluation on an NVIDIA Tesla P100 GPU with 16 GB memory.

As for the evaluation, following previous work, we use the popular natural language processing metrics BLEU,^[^ ^24^ ^]^ METEOR,^[^ ^25^ ^]^ ROUGE_L,^[^ ^26^ ^]^ CIDEr^[^ ^27^ ^]^ for the performance on difference question answering. For the zero‐shot prediction provided by the LLMs, we report the performance of a single run due to budget constraints. For other question types, we adhered to the common practice of using accuracy as the evaluation metric.

## Conflict of Interest

The authors declare no conflict of interest.

## Author Contributions

**Zishan Gu**: conceptualization (lead); formal analysis (lead); methodology (lead); writing—original draft (lead); writing—review and editing (lead). **Fenglin Liu**: conceptualization (supporting); formal analysis (supporting); methodology (supporting); writing—review and editing (supporting). **Jiayuan Chen**: formal analysis (supporting); validation (supporting); writing—review and editing (supporting). **Changchang Yin**: conceptualization (supporting); methodology (supporting); writing—review and editing (supporting). **Ping Zhang**: conceptualization (supporting); funding acquisition (lead); writing—review and editing (supporting).

## Supporting information

Supplementary Material

## Data Availability

The data that support the findings of this study are openly available in PhysioNet at https://physionet.org/content/medical‐diff‐vqa/1.0.0/, reference number 100.
