# Supplementary material for: A Proactive Agent Collaborative Framework for Zero‐Shot Multimodal Medical Reasoning
Source: Adv Intell Syst. 2025 Feb 5;7(8):2400840. doi: 10.1002/aisy.202400840 (PMC12370165; doi:10.1002/aisy.202400840)
Supplement: Supplementary file 1 — Supplementary Material [file AISY-7-0-s001.pdf]

Supplementray Information for
Inquire, Interact, and Integrate: A Proactive Agent Collaborative Framework for Zero-Shot Multimodal Medical Reasoning

A Dataset Statistics

Question Type	Example	# QA pairs	# answer candidates
Abnormality	what abnormalities are seen in this image?	59,435	8316
Abnormality*	what abnormalities are seen in the upper lungs?	85,986	25
Presence	is there evidence of atelectasis in this image?<br>Is there edema?	155,726	2
View	which view is this image taken?<br>is this AP view?	56,265	4
Location	where in the image is the pleural effusion located?<br>is the atelectasis located on the left side or right side?	84,193	735
Type	what type is the opacity?<br>what type is the atelectasis?	27,478	87
Level	what level is the cardiomegaly?<br>what level is the pneumothorax?	67,296	112
All	-	536,379	9,256
Difference	what has changed compared to the reference image?<br>what has changed in the right lung area?	164,324	-

Table S1. Statistics of the MIMIC-CXR-VQA dataset. *Abnormality** stands for the abnormality questions excluding the question “what abnormalities are seen in this image?”. *All* stands for the questions excluding the difference type of questions. Note that the examples shown here are the learning context we provide to the learner LLM models.

B Hyperparameter Study of Domain Expert Models

We have performed preliminary experiments with model MMQ on abnormality type of questions, and the experimental results are demonstrated in Table S2. It can be observed that a feature dimension of 64 and a learning rate of 0.01 slightly outperformed the other configurations.

Feature Dimension	Learning Rate	Accuracy
32	0.001<br>0.005<br>0.01	0.7453<br>0.7324<br>0.7511
64	0.001<br>0.005<br>0.01	0.7488<br>0.7592<br>**0.7671**
128	0.001<br>0.005<br>0.01	0.7459<br>0.7583<br>0.7233

Table S2. Performance comparison of MMQ model on abnormality-type questions.

C VQA Performance

We present the performance of various VQA models on single-image-related questions in Table S3. It can be observed that employing a divide-and-conquer strategy significantly improves performance, thereby enabling our MultiMedRes to acquire accurate information throughout the communication process.

Models	Open	Close	All
MMQ	11.5	10.8	11.2
EKAID	26.4	79.9	52.5
UIO	0.04	53.6	26.2
MiniGPT-v2	15.5	57.3	35.9
LLaVa	13.1	55.8	33.9
LLaVa-Med	21.1	52.8	36.6
LLM-CXR	24.3	55.8	39.7
XrayGPT	3.87	41.2	22.1
Ours	**54.3**	**84.8**	**69.2**

Table S3. Comparative performance of various models on questions concerning single images.

D Rectifying Effect of Learner Agent

We present a case study where a learner agent rectifies errors made by domain experts. Specifically, the learner agent doubts the existence of vascular congestion in the main image and suspects cardiomegaly in the reference image, diagnoses previously provided by the experts. Through a focused interaction with the experts using yes-or-no questions targeting specific abnormalities, the experts refine their answers, leading to a more accurate final summarization of the differences.

E Prompts

We exhibit two example prompts for GPT and LLaMa 2 in Figure S2 and Figure S3 respectively. We change the difference questions (highlighted in blue) accordingly at runtime.

We also demonstrate the prompt for LLaVa with and without our generated dialogue.

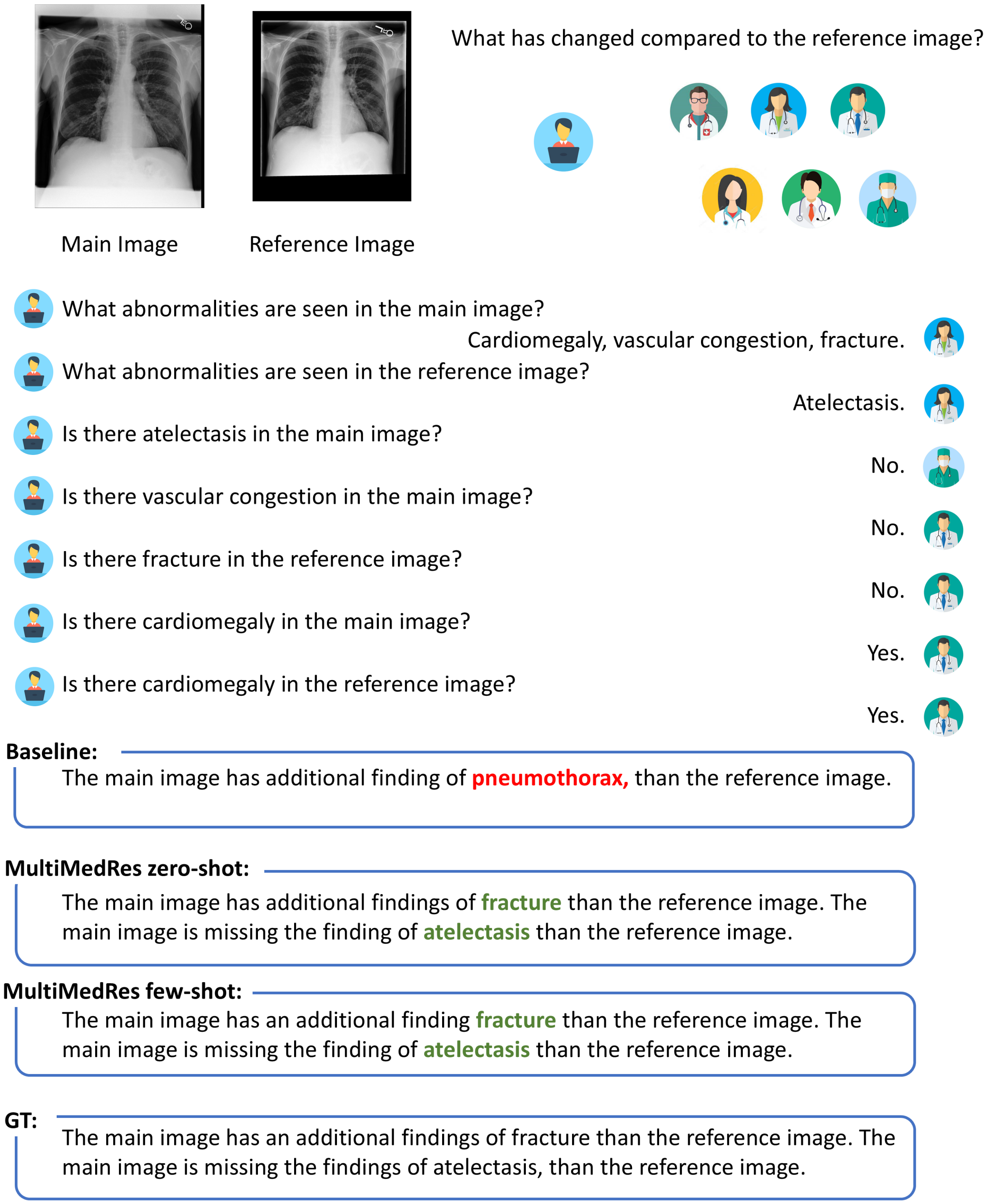

Figure S1. Rectifying Effect of Learner Agent.

Example Prompt for GPT

```
You are a radiologist trying to answer questions that pertain to the clinical progress and changes in
the main image as compared to the reference image. I will give you example answers in the format of
question-answer pairs.

'what has changed compared to the reference image? the main image has an additional finding of
pneumothorax than the reference image. the main image is missing the findings of fracture, lung
opacity, and pleural effusion than the reference image.'
'what has changed compared to the reference image? the main image is missing the findings of lung
opacity, consolidation, pleural effusion, and pleural thickening than the reference image.'
'what has changed in the right lung area? the level of pleural effusion has changed from small to
moderate.'
'what has changed compared to the reference image? nothing has changed.'

You can ask questions about both images to gather information.
These are six types of questions you can ask.

You can ask about the abnormalities in an image with the key being 'abnormality', like 'what
abnormalities are seen in this image?', 'what abnormalities are seen in the upper lungs?'.
You can ask about the presence of a certain abnormality in an image with the key being 'presence',
like 'is there evidence of atelectasis in this image?', 'is there edema?'.
You can ask about the level of a certain abnormality in an image with the key being 'level', like
'what level is the cardiomegaly?', 'what level is the pneumothorax?'.
You can ask about the location of a certain abnormality in an image with the key being 'location',
like 'where in the image is the pleural effusion located?', 'is the atelectasis located on the left
side or right side?'.
You can ask about the type of a certain abnormality in an image with the key being 'type', like 'what
type is the opacity?', 'what type is the atelectasis?'.
You can ask about the view of an image with the key being 'view', like 'which view is this image
taken?', 'is this AP view?'.


Give me your questions one at a time about any of the images in the format of a Python dictionary with
keys recording the imageID, question_type and question_content. Only return the Python dictionary.
In order answer this question for a main image with ID 000A with reference image ID 000B: what has
changed compare to the reference image? You can start asking questions now.

Do not ask repeated questions, and ask as less questions as possible. You should stop asking questions
once you have enough information.
Only reply with the difference when you answer the question. No explanation is needed.
```

Figure S2. An example of the prompt for GPT.

Example Prompt for LLaMa 2

System Prompt:

You are a helpful radiologist assistant trying to answer questions that pertain to the clinical progress and changes in the main image as compared to the reference image. I will give you example answers in the format of question-answer pairs.

'what has changed compared to the reference image? the main image has an additional finding of cardiomegaly than the reference image.
'what has changed compared to the reference image? the main image is missing the finding of lung opacity than the reference image.'
'what has changed compared to the reference image? the main image has additional findings of cardiomegaly, and vascular congestion than the reference image. the main image is missing the findings of pleural thickening than the reference image. '
'what has changed in the right lung area? the level of pleural effusion has changed from small to moderate.'
'what has changed compared to the reference image? nothing has changed.'

In order to answer the question like these examples, you can ask questions about both images to gather information.

You can ask about the abnormalities in different images with the question_type being 'abnormality', like 'what abnormalities are seen in this image?', 'what abnormalities are seen in the upper lungs?'.
You can ask about the presence of a certain abnormality in an image with the question_type being 'presence', like 'is there evidence of atelectasis in this image?', 'is there edema?'.
You can ask about the level of a certain abnormality in an image with the question_type being 'level', like 'what level is the cardiomegaly?', 'what level is the pneumothorax?'.
You can ask about the location of a certain abnormality in a image with the key being 'location', like 'where in the image is the pleural effusion located?', 'is the atelectasis located on the left side or right side?'.
You can ask about the type of a certain abnormality in a image with the key being 'type', like 'what type is the opacity?', 'what type is the atelectasis?'.
You can ask about the view of an image with the key being view', like 'which view is this image taken?', 'is this AP view?'.

Give me your questions one at a time about any of the images in the format of a Python dictionary with keys recording the imageID, question_type and question_content. Only return the Python dictionary. You can only ask about the 'abnormality', the 'presence', the 'level', the 'location', the 'type' or the 'view'.

Initial Prompt:

[INST] In order to answer this question for a main image with ID 000A with reference image ID 000B: what has changed compare to the reference image? You can start asking questions about both images now. You should stop asking questions once you have enough information and, starting with the keyword 'Answer:', only reply with the difference when you answer the question. Please follow the format of the provided question-answer pairs. No explanation is needed. [/INST].

Figure S3. An example of the prompt for LLaMa.

Example Prompt for LLaVa

System Prompt:

A chat between a curious user and a radiologist assistant.
The assistant is able to answer questions that pertain to the clinical progress and changes in the main image on the left as compared to the reference image on the right.
The assistant will only reply with the answer according to the following examples of question-answer pairs.
'what has changed compared to the reference image? the main image has an additional finding of pneumothorax than the reference image. the main image is missing the findings of fracture, lung opacity, and pleural effusion than the reference image.',
'what has changed compared to the reference image? the main image is missing the findings of lung opacity, consolidation, pleural effusion, and pleural thickening than the reference image.',
'what has changed in the right lung area? the level of pleural effusion has changed from small to moderate.'
'what has changed compared to the reference image? nothing has changed.'
The visual content will be provided with the following format: <Image>visual content</Image>.

Prompt:

USER: <im_start><image><im_end>
Given the main image on the left and the reference image on the right: what has changed compare to the reference image?
ASSISTANT:

Prompt with dialogue:

USER: <im_start><image><im_end>
Here are several rounds of communication between a learner and specialists discussing the images.
<MultiMedRes Chatlog>
Now, given the main image on the left and the reference image on the right: what has changed compare to the reference image?
ASSISTANT:

Figure S4. An example of the prompt for LLaVA.
